# MicroRNAs: regulators of oncogenesis and stemness

**DOI:** 10.1186/1741-7015-6-15

**Published:** 2008-06-24

**Authors:** Thales Papagiannakopoulos, Kenneth S Kosik

**Affiliations:** 1 Neuroscience Research Institute, University of California, Santa Barbara, Santa Barbara, CA 93106, USA; 2 Department of Molecular, Cellular and Developmental Biology, University of California, Santa Barbara, Santa Barbara, CA 93106, USA

## Abstract

MicroRNAs (miRNAs) are essential post-transcriptional regulators that determine cell identity and fate. Aberrant expression of miRNAs can lead to diseases, including cancer. Expression of many miRNAs in the de-differentiated brain tumor cancer stem cells resembles that of neural stem cells. In this issue of *BMC Medicine*, Silber et al provide evidence of the expression of such miRNAs and their potential to mediate differentiation in both stem cell populations. In this commentary, we discuss the known functions of miRNAs in cancer and stem cells, their therapeutic potential and how the findings of Silber et al provide insight into the role of miR-124/miR-137 dysregulation in glioblastomas.

## MicroRNA biogenesis and function

**Figure 1:**
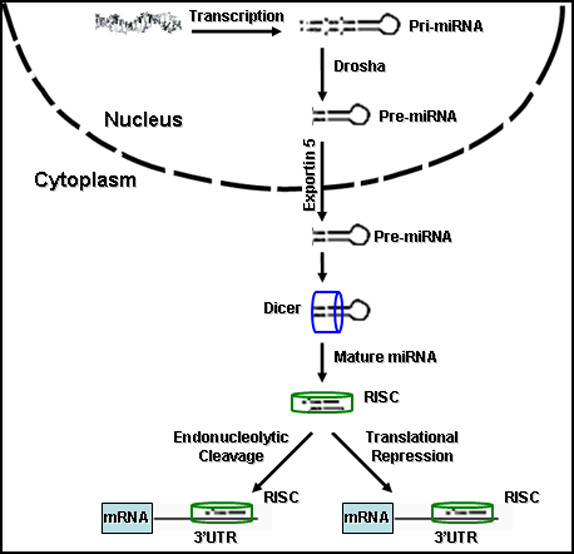
**MicroRNA biogenesis**. Primary transcripts are transcribed from the genome and form a long hairpin loop, pri-miRNA, which is processed by Drosha to form pre-miRNA. Pre-miRNA is exported to the cytoplasm by Exportin 5 where Dicer cleaves off the hairpin loop to form a duplex that contains the mature 21–23 nucleotide microRNA (miRNA). The mature miRNA is then incorporated into the RNA-induced silencing complex to target the 3' untranslated region of the target mRNA.

With over 500 microRNA (miRNA) genes identified experimentally in the human genome and a plethora of computationally predicted mRNA targets, it is believed that these small non-coding RNAs have a central role in diverse cellular and developmental processes. miRNAs function as endogenous triggers of the RNA interference pathway. When mature (active) they are ~22 nt small RNAs, generated by two cleavage steps, which are catalyzed by two distinct complexes: Drosha, which is found in the nucleus, and Dicer, which is found in the cytoplasm (Figure 1) [1]. Following Dicer cleavage, the mature form of the miRNA is incorporated into the RNA-induced silencing complex (RISC) where the 3' untranslated region (3'-UTR) of the target mRNA associates with the major component of the RISC complex, Argonaute (Figure 1) [1].

miRNAs act on cellular transcripts by two different mechanisms. The first mechanism is cleavage-dependent RNA degradation of the transcript at the site where the miRNA binds. The other mechanism is miRNA-mediated translational repression of the target transcript, while the miRNA binds to the target 3'-UTR with several mismatches. This partial complementarity allows for miRNAs to target multiple 3'-UTRs [1]. A key question that arises from the targeting of multiple transcripts is whether there has been evolutionary enrichment in targets that are involved in similar cellular functions.

## miRNAs in cancer

Aberrant expression of miRNA genes can lead to human diseases, including cancer. In recent years, a number of informative profiling studies have determined the levels of miRNAs in various types of cancer cell lines and tumors [2]. Most of these studies have identified multiple dysregulated miRNAs, which in many cases can classify cancer types [3]. In addition, multiple functional studies on tumor suppressive or oncogenic miRNAs were identified from profiling, screens or due to their cancer-associated genomic loci [2,3].

The first tumor suppressive miRNAs identified were the *miR-15/miR-16 *cluster, located in a genomic region of chr13, which is often deleted in chronic lymphocytic leukemias (CLLs). Therefore, these two miRNAs are not expressed in CLLs, leading to increased levels of an oncogenic target, the apoptotic inhibitor Bcl-2 [2].

The first functional evidence of a miRNA, or a non-coding RNA, acting as a mammalian 'oncogene' or 'onco-miR' was the miR-17–92 polycistron. Enforced expression of this 'onco-miR' was shown to act with c-Myc to accelerate tumor development in a mouse B-cell lymphoma model [4]. c-Myc, which is a well-studied oncogene, activates the expression of *miR-17–92 *through its direct interaction with the putative promoter region. This finding could explain the high expression levels of the miRNA cluster in lung, breast, stomach, prostate, colon and pancreatic cancers where it potentially plays a similar oncogenic role [2,5]. miRNAs from this cluster target tumor suppressor genes such as *E2F1*, *PTEN *and *TGFβRII *[2,5].

Gliomas are the most common primary tumors in the brain and are divided into four clinical grades on the basis of their histology and prognosis. Grade IV Glioblastomas are the most malignant of all brain tumors and are almost always fatal [6,7]. Profiling of glioblastoma tumors and cell lines has revealed increased levels of miR-21 [8]. In the same study, inhibition of miR-21 in glioblastoma cells led to increased cell death, suggesting that miR-21 could be acting as an oncogene to inhibit cell death in glioblastomas. Subsequent cancer profiling studies also reported increased levels of miR-21 in most types of cancer where miR-21 had the same anti-apoptotic role [2]. Recently, a study determined that miR-21 knockdown in glioblastoma cells significantly repressed the ability of these cells to form tumors in an *in vivo *mouse xenograft model [9].

In this issue of *BMC Medicine*, a study by Silber et al demonstrates that two miRNAs, miR-124 and miR-137, are significantly decreased in anaplastic astrocytomas (AA, WHO grade III) and glioblatoma multiforme (GBM, WHO grade IV) relative to non-neoplastic brain tissue. In addition to the profiling performed in this study, *in situ *hybridization experiments would be helpful to determine the exact cell populations in which miR-124 and miR-137 are dysregulated. When these miRNAs were overexpressed in glioblastoma cell lines, they led to G1 cell cycle arrest by repression of Retinoblastoma (RB) and CDK6. Downregulation of CDK6 occurs partially through direct targeting by miR-137. This is a significant tumor suppressive potential of these two miRNAs by repressing cell cycle progression and thus the growth of GBM cells.

## microRNAs in cancer stem cells

Chen et al were the first to suggest that embryonic stem cells (ESCs), embryoid bodies, and day 11 mouse embryos exhibit a less complex miRNA profile than more mature somatic tissues. They hypothesized that the degree of cellular or tissue differentiation can be characterized by a particular miRNA signature [10]. The obvious parallel that can be drawn is that undifferentiated stem cells display miRNA expression profiles reminiscent of cancer cells. Numerous known oncogenic miRNAs are expressed in early stages of development in undifferentiated cells; however, their expression decreases in differentiated tissue, whereas the opposite holds true for tumor suppressive miRNAs.

In recent years, there has been a sudden increase in studies on a subpopulation of cells in tumors that have stem cell properties, cancer stem cells (CSCs). The CSC hypothesis proposes that cancers are derived from a small fraction of cancer cells that constitute a reservoir of self-sustaining cells with the exclusive ability to self-renew and maintain the tumor. Small numbers of CSCs are sufficient to form orthotopic tumors *in vivo*. Cells which could be described as CSCs have been isolated from hematological malignancies, brain, breast and colon tumors [6,7,11-13].

GBM-derived CSCs share the fundamental stem cell properties of self-renewal and multi-potency with neural stem cells (NSCs). They were first isolated from tumors by virtue of the expression of CD133 that marks NSCs and neural progenitor cells (NPCs) [6,7]. Microarray studies have since revealed considerable overlap between the gene-expression signatures of glioblastomas and progenitor cells of the developing forebrain. Forced differentiation of CD133^+ ^cells with BMP4 addition abrogates their stem cell phenotypes, making this a potential therapeutic approach for eliminating this subpopulation of GBMs [7].

**Figure 2:**
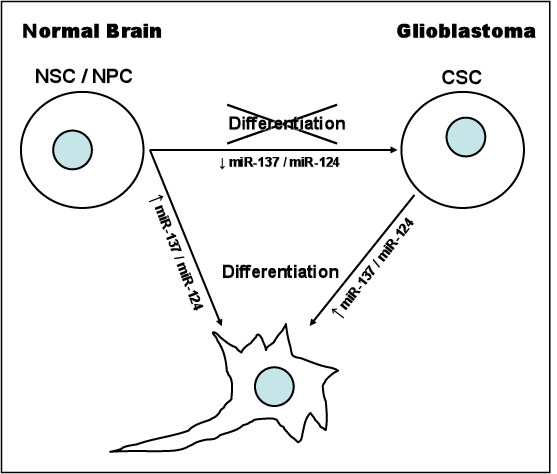
**mir-137 and mir-124 are essential for the canonical differentiation of neural stems cells and neural progenitor cells**. Abrogation of their expression could lead to cancer stem cell (CSC) formation and tumorogenesis. Upregulation of these two microRNAs in CSCs could present a therapeutic approach in differentiating these cells, thus eliminating their stem cell properties.

In the study published by Silber et al, two miRNAs with low expression in GBMs and AAs, miR-124 and miR-137, increase in expression upon differentiation of mouse NSCs with growth factor withdrawal (Figure 2). Interestingly, the expression of miR-124, which is a brain enriched miRNA, has been shown to correlate with the first appearance of differentiated neurons in culture from NPCs [14]. Overexpression of these two miRNAs in undifferentiated mNSCs, mouse oligodendroglioma-derived stem cells and CSCs termed human GBM-derived stem cells (hGSCs) led to morphological changes and expression of markers indicating neuronal differentiation (Figure 2). The forced differentiation of these CSCs which is mediated by these two miRNAs leads to loss of their self-renewal and oncogenic capacity. These data are in accordance with previous approaches where CSC oncogenicity was severely disrupted by differentiation. A challenge in this field is to define the functional, molecular, regulatory and antigenic features that characterize NPCs from CSCs and ultimately to devise therapeutic strategies that specifically target the tumor population, but leave normal stem and precursor cells unharmed.

Treatment of GBM tumors with current methods has proven largely unsuccessful. To a great extent this is because of the ineffectiveness of current therapeutics to target the CSC population of GBM tumors. This study indicates a new approach targeting the CSC subpopulation of tumors by overexpressing microRNAs which are normally expressed in the surrounding differentiated tissue. Upregulation of miR-124 and miR-137 could have therapeutic use in blocking this CSC subpopulation of GBM tumors. Modulation of GBM tumor dysregulated miRNAs such as miR-21 and miR-124/miR-137 by RNAi-based therapeutics, in conjunction with current treatments may prove a key approach in the fight against these deadly tumors.

Development of miRNA/RNAi-based therapeutics requires several critical experimental steps, which include: (1) miRNA profiling of cancer versus healthy tissue, (2) functional analysis of dyregulated miRNAs, and (3) *in vivo *studies with use of different RNAi-based therapeutic methods to dysregulate miRNAs. Silber et al have performed the two initial steps for miR-124/miR-137 as therapeutic targets in GBMs. A major caveat in RNAi-based therapeutics has been their *in vivo *targeting to the brain and particularly crossing of the blood brain barrier [15]. This problem can be overcome with the use of lipid encapsulation, direct administration of the therapeutics to the brain tumor or development of safe gene therapy approaches using viral delivery [15]. In the dawn of miRNA research, there is confidence that RNAi can be successfully maintained *in vivo *in a tumor-specific manner for prolonged clinical efficacy. The success of such strategies for gene therapy will provide clinicians with a larger repertoire that includes miRNA-therapeutic agents, which can be used in parallel with currently available drugs.

## Pre-publication history

The pre-publication history for this paper can be accessed here:


